# Modified bathroom scale and balance assessment: a comparison with clinical tests

**DOI:** 10.1186/s40064-016-2086-8

**Published:** 2016-04-18

**Authors:** Jacques Duchêne, David Hewson, Pierre Rumeau

**Affiliations:** Institut Charles Delaunay, UMR CNRS 6279, University of Technology of Troyes, 12 Rue Marie Curie, CS 42060, 10004 Troyes, France; Institute for Health Research, University of Bedfordshire, University Square, Luton, Bedfordshire LU1 3JU UK; Department of Geriatrics, Gérontopôle, 170, Avenue de Casselardit, TSA 40031, 31059 Toulouse Cedex, France

**Keywords:** Modified bathroom scale, Fall risk, Frailty, Modelling, Prevention

## Abstract

Frailty and detection of fall risk are major issues in preventive gerontology. A simple tool frequently used in daily life, a bathroom scale (balance quality tester: BQT), was modified to obtain information on the balance of 84 outpatients consulting at a geriatric clinic. The results computed from the BQT were compared to the values of three geriatric tests that are widely used either to detect a fall risk or frailty (timed get up and go: TUG; 10 m walking speed: WS; walking time: WT; one-leg stand: OS). The BQT calculates four parameters that are then scored and weighted, thus creating an overall indicator of balance quality. Raw data, partial scores and the global score were compared with the results of the three geriatric tests. The WT values had the highest correlation with BQT raw data (r = 0.55), while TUG (r = 0.53) and WS (r = 0.56) had the highest correlation with BQT partial scores. ROC curves for OS cut-off values (4 and 5 s) were produced, with the best results obtained for a 5 s cut-off, both with the partial scores combined using Fisher’s combination (specificity 85 %: <0.11, sensitivity 85 %: >0.48), and with the empirical score (specificity 85 %: <7, sensitivity 85 %: >8). A BQT empirical score of less than seven can detect fall risk in a community dwelling population.

## Introduction

Balance is essential in order to perform typical activities of daily living. However, the ability of people to maintain their balance decreases with age and/or neurological or musculoskeletal disorders. Older adults with poor balance have an increased risk of falls and adverse health outcomes (Fried et al. 2001), and also reduced physical activity levels, leading to an increased risk of frailty (Fried et al. 2005) and a decline towards dependence (Tinetti and Williams 1997; Gill et al. 1995). Despite this gradual decline, it is possible to slow down or even prevent the decline for community-dwelling elderly if an appropriate intervention program is put in place (Gill et al. 2002). In order to be effective, the intervention program needs to start as early as possible, which means there is a need for tools that are able to detect as early as possible any decrease in balance quality. Thus, an adapted prevention or rehabilitation program could be put in place, with progress followed over time. Accordingly, it is essential to detect balance impairment as early as possible, and then to monitor balance quality under ecologically valid conditions. Any evaluation tool needs to be easy to use (non professional users), socially acceptable (community dwelling elderly) and relevant with respect to well-established clinical tests.

Balance quality is typically measured under controlled conditions such as a research laboratory using force plates, which provide measures based on the centre of pressure (CoP) displacement. Such measures are considered to be the gold standard measure of balance, with these tests providing comparable results with those of clinical balance tests (Haas and Burden 2000; Berg et al. 1992). However, force plates are expensive and cannot be considered for home use, or even routine clinical practice.

**Fig. 1:**
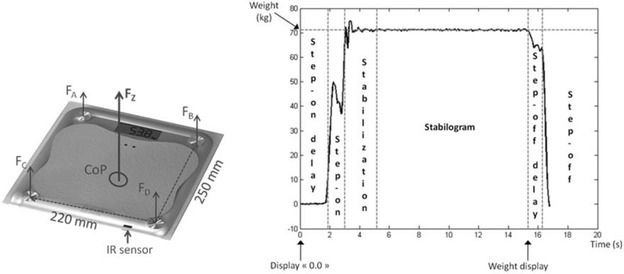
**a** The BQT, with the forces measured and the CoP calculated; **b** a typical recording of the BQT for an older adult showing the period where each parameter is calculated

A new device has been developed that can overcome the problems identified for home use of force plates, while still providing accurate measurement of balance quality. This device, which is a modified bathroom scale (balance quality tester: BQT), acts like a force plate, but still looks like a bathroom scale (Duchêne and Hewson 2011). The BQT is based on a standard scale, with some modifications in order to collect the raw data produced by each of the four sensors of the scale. The instantaneous vertical force (Fz) is then reconstructed as the sum of the forces measured by each sensor, while the position of the CoP can be estimated as the barycentre of these four forces. The current version of the scale and an illustration of the forces and CoP can be seen in Fig. 1. A detailed description of the BQT can be found in (Duchêne and Hewson 2011).

A large number of clinical tests are available to measure balance in older adults. For detailed reviews and comparisons, see the comprehensive review of Langley and Mackintosh (2007), that identified 17 different clinical tests to measure balance, and the comparison of Mancini and Horak (2010) between different clinical and objective tests. Among all these tests, several are widely used in clinical practice to assess balance, namely: the Berg balance test (Berg and Norman 1996; Berg et al. 1992), which focuses specifically on balance impairment; the Performance Oriented Mobility Assessment (POMA) test, which includes both balance and gait walking tasks (Tinetti 1986); and the timed up and go (TUG) test (Mathias et al. 1986), which has been described as a good predictor of the falls in community-dwelling older adults (Shumway-Cook et al. 2000).

An initial attempt to compare the results of the BQT and the three clinical tests outlined above was performed with two groups of older adults, the first of which was living in nursing homes, while the second was a group living in the community (Vermeulen et al. 2012). Although these results suggested that the BQT was a useful tool for measuring balance in older adults, the added value of the BQT in clinical practice remained to be demonstrated. In addition, this work made use of an empirical score first defined in (Duchêne and Hewson 2011), which, although able to discriminate between the two groups in the study, has not been optimized with respect to a reference objective.

The aim of the present study was to evaluate the external validity of the BQT under clinical conditions to detect fall risk. To this end, three clinical tests, two of which were not used in the previous work, were used: the TUG test (Mathias et al. 1986), the walking speed (WS) test (van Kan et al. 2009), and the one-leg stand (OS) (Vellas et al. 1997), with cut-off values for fall risk already identified for the last two tests:

- 6 or 7 s for a 4.57 m walk, depending on height and gender, which is one of Fried’s five indices discriminating between frail and not frail (Fried et al. 2001)
- 5 s for the OS, (Vellas et al. 1997), which discriminates between people at risk of falls or not.

The use of tests with cut-offs offers the possibility of assessing the sensitivity and specificity of the score of the BQT with respect to an objective of risk of falls.

## Methods

### Balance quality tester

The protocol for using the BQT is straightforward, with an in-depth description provided in (Duchêne and Hewson 2011):

- Stand in front of the BQT, whereby an infrared detector detects the presence of the person
- Wait for the scale to display “0.0”
- Step onto the scale
- Wait for the scale to display body weight
- Step off the scale.

In the previous works using the BQT (Duchêne and Hewson 2011; Vermeulen et al. 2012), four variables were computed and scaled to construct a global score: delay before stepping onto the BQT after “0.0” has been displayed, rate of stepping onto the BQT, the surface area of the CoP displacement, and the speed of CoP displacement. The first variable (delay) is only of use when people are self-tested, such as in their own homes without someone present to instruct them to step onto the BQT. In home testing the delay before stepping onto the BQT could be considered to be a measure of the hesitancy of the user to step onto the BQT. However, in a clinical environment, the user is requested to step onto the BQT by a clinician, thus rendering the delay parameter of no use. Accordingly, the delay before stepping on was replaced by another parameter, namely the coefficient of variation of the vertical force Fz during the stabilization stage. This parameter was calculated during a 2-s window once the subject had stepped onto the device. Finally, the scale of the trajectory velocity has been modified from the previous version, without affecting the values used in previous studies. Detailed calculations of the four parameters can be found in “Appendix”. Figure 1 shows an example of a typical recording, with the periods where each parameter is calculated. A summary of the scoring for the four parameters as well as the global score is shown in Table 1.

**Table 1:** Scoring of the four parameters in the BQT score

Score value	Coefficient of variation (%)	Rising rate (kg s^−1^)	Stabilogram surface (cm^2^)	Trajectory velocity (cm s^−1^)
0	≥5.5	<60	≥12	≥5
1	4.5, <5.5	60, <80	8, <12	4, <5
2	3.5, <4.5	80, <100	5, <8	2.5, <4
3	2.5, <3.5	100, <120	3, <5	2, <2.5
4	<2.5	≥120	<3	<2

### Protocol design

Eighty-four participants (27 men, aged 81.5 ± 7.5 years; 57 women, aged 84.0 ± 5.5 years) were recruited among patients coming for a geriatric examination at the Toulouse University Hospital (CHU Toulouse, France) from June 2009 to July 2011. The criteria for inclusion were an age over 65 years, and to be capable to step onto the BQT. People with severe handicaps, acute pathologies or current treatment for physical injury were excluded from the experiment. All participants volunteered and signed an informed consent. The protocol was approved by a Regional Ethical Committee (CCPPRB ref: 2007-A00320-53, date: 2007-05-24).

All participants had a geriatric evaluation, which included the TUG, WS, and OS, with the latter taken as clinical reference test for BQT validation in respect to fall risk. A range of other tests were also performed as part of the geriatric evaluation, such as the GDS, MMS, Tinetti, and Stop Walking when talking, but these tests are not presented in the present paper.

Each participant followed the protocol described above when using the BQT. Subjects performed three repetitions of the BQT test, with the score calculated from the mean of each variable, estimated from all validated repetitions achieved within the same session (tests were validated by the clinician when all steps of the protocol were respected).

In addition to the population of older adults, 20 control subjects (10 men and 10 women) recruited within the university were also tested (aged 28.8 ± 9.4 year). The control subjects were tested using the BQT as well as for the OS, with this test stopped if subjects reached 15 s of single leg stance. The number of control subjects was not matched to the older subjects tested, as the aim of the control group testing was to validate the maximal score of the BQT.

### BQT score

In previous work, the overall BQT balance score was computed in an empirical manner, by adding up the partial scores produced by each of the variables extracted from the BQT raw data. The native variables (NV) were all given the same weighting in the overall balance score, with this original score refered to as the empirical score (ES). Although acceptable results were found, such an arbitrary score might not have been the best representation of balance. In the present study both the partial scores (PS) and the NV were weighted in order to optimize the correlation with each of the clinical tests (TUG, WS and OS). Regression models were constructed for each of the three clinical tests and each of the three types of data (NV, PS, and ES). Models were only for computed for the clinical tests that had a normal distribution.

### Data analysis

As indicated above, prior to multiple regression analysis, the Gaussian nature of the observed variables was tested using the Kolmogorov–Smirnov statistical test (Dudewicz and Mishra 1988). Correlations were then computed between the normally distributed clinical tests and the various outputs obtained from the linear modelling process. Regression coefficients were computed using the “regress” function available in MATLAB^®^ (Mathworks Inc, Natick, MA, USA). The correlation coefficient was obtained from the R^2^ statistic (square of the correlation coefficient R) produced by the same MATLAB^®^ function.

In the second step of the data analysis, an estimation of the sensitivity and specificity of ES, or an optimized combination of PS, was computed in relation to fall risk. In this case, performance in the OS was taken as fall-risk, with subjects classified with respect to the 5-s cut-off value. receiver-operator characteristic (ROC) curves were used to express sensitivity (vertical axis) and specificity (horizontal axis) of the variables to classify subjects as at risk, or not at risk, of falling (Zweig and Campbell 1993). In addition the area S under the ROC curve, which can be taken as a global index of the accuracy of the classification, was used to compare different conditions in terms of classification accuracy (Hanley and McNeil 1982). The optimized combination of PS was obtained with respect to the best linear classification, with Fisher’s linear discriminant function used (Fisher 1936).

## Results

### Normality of the data

**Fig. 2:**
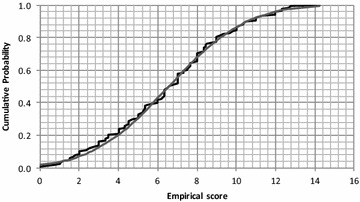
Distribution of ES in comparison with the normal distribution

The Kolmogorov–Smirnov Dmax distances were 0.134, 0.130 and 0.274 for the distributions of the TUG, WS, OS tests, respectively. For N = 84, the critical value of Dmax is 0.146 (p = 0.05), meaning that only TUG and WS could be considered to have normal distributions. Therefore, multiple regression analysis was conducted only for these two tests, with OS being used for subsequent classification in respect fall risk. The same test was also used for ES, with the corresponding Kolmogorov–Smirnov Dmax of 0.057, which indicates that ES has a highly normal distribution (Fig. 2).

### Regression analysis

Walking speed was calculated from a measure of the time to walk 10 m (walking tine: WT), which is a non-linear transformation. Given that linear modelling was used, both WS and WT were analysed, as well as TUG. The modelling process produced regression coefficients, as well as the coefficient of determination R^2^ and the corresponding F-statistics. The results of the clinical tests, including the correlation between the observed variables and ES, are shown in Table 2, with p values for all models <0.0001.

**Table 2:** Linear regression results

Clinical test	BQT native variables			BQT partial scores			Empirical scores		
	R	R^2^	F	R	R^2^	F	R	R^2^	F
TUG	0.52	0.27	7.36	0.53	0.28	7.60	0.50	0.25	27.81
WT	0.55	0.30	8.47	0.52	0.27	7.46	0.50	0.25	27.16
WS	0.52	0.27	7.40	0.56	0.31	8.96	0.54	0.29	34.19

**Fig. 3:**
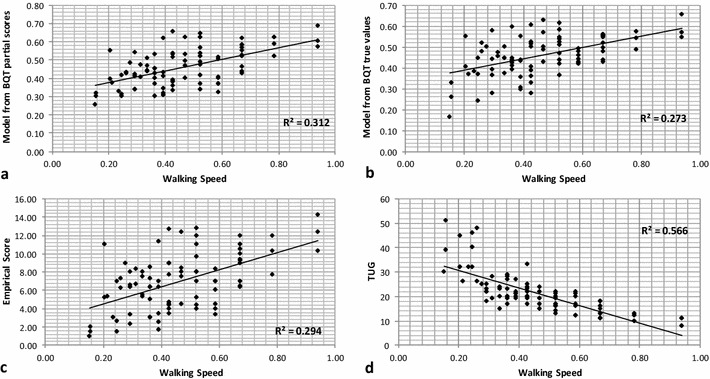
**a** WS versus BQT PS; **b** WS versus BQT NV; **c** WS versus ES; **d** WS versus TUG

The result for the model with the best fit, WS against BQT PS, is shown in Fig. 3a, while WS against the BQT NV is shown in Fig. 3b. The relationship between WS and ES is shown in Fig. 3c, while the correlation between the two observed variables WS and TUG can be seen in Fig. 3d.

### Classification performance

Classification was conducted in respect to fall risk classified using the OS test with a 5-s cut-off (Vellas et al. 1997). Fisher’s coefficients were obtained after normalization, with clinical tests centred and divided by their standard deviation. Better results were obtained for PS than for NV, with discrimination ratios of λ = 33 % and λ = 22.5 % for PS and NV, respectively.

The weightings obtained for the individual variables were 0.61 for the trajectory velocity, 0.57 for the coefficient of variation, 0.42 for the surface area, and 0.36 for the rise rate. Classification using ES, which had the same weighting for all PS, produced a discrimination ration of λ = 25.5 %.

**Fig. 4:**
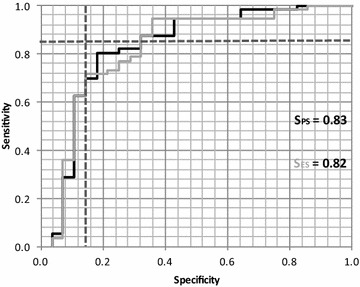
ROC curves obtained for ES (*grey tracing*) and for PS after projection on the Fisher discriminant axis (*black tracing*). *Horizontal and vertical dashed lines* represent 85 % of true and 15 % of false positives, respectively

The ROC curves for PS and ES are displayed in Fig. 4, where S_PS_ and S_ES_ represent the surface under PS and ES curves, respectively.

Fisher’s combination of PS and ES for the limits of 85 % in sensitivity and specificity are shown in Table 3.

**Table 3:** ROC curve limits for 85 % sensitivity and specificity

	Fisher’s combination of partial scores	Empirical score
Specificity 85 %	<0.11	<7
Sensitivity 85 %	>0.48	>8

### Control subjects

None of the control subjects obtained an ES of <15 out of a maximum score of 16, with all subjects able to stand on one leg for at least 15 s. For those subjects for whom the score was 15, (9 out of 20 subjects), the variable that reduced the score was the trajectory velocity in 78 % of cases (7 out of 9 subjects).

## Discussion

### General issues

In the present study results were presented in two ways. Firstly, in respect to how the BQT scores compare to standard clinical tests, and secondly, the accuracy with which the BQT can classify fall risk, in reference to one-leg stand time.

Slower walking speed as a single variable has been shown to predict subsequent adverse events (Montero-Odasso et al. 2005), with this variable forming one of the five indices of frailty proposed by Fried et al. (2001). Several studies have suggested that walking speed at preferred velocity might be the best single factor in predicting frailty (Theou et al. 2011; Mitnitski et al. 2001). Similar results were observed in the present study, with a high correlation found between the TUG and WS (r = 0.75), and the TUG and WT (r = 0.80). The higher correlation might be due to the non-linear nature of the transformation from time to velocity. Although the BQT does not measure the same physical capacity as the WS test, significant correlations between the results of both tests were observed. The best correlation was obtained between WS and the optimal combination of PS (r = 0.55). The fact that the raw values of the variables do not produce the best performance can be explained by the non-linear transformation from true values towards the corresponding PS. It should be noted, however, that the ES, which is a simple addition of all PS, had a correlation that did not differ significantly from that obtained for the optimal combination (r = 0.54).

For the classification performance, only partial and ES were considered. The OS test was not considered for correlation analyses, due to the highly non-normal nature of the data produced by this test. Such a finding was expected, as the nature of this and any test where the time for which an action can be performed tends to produce a skewed distribution. Despite this lack of normality, the OS has been shown to accurately classify people at risk of falling (Vellas et al. 1997). In addition, the cut-off for the OS test is independent of the anthropometric characteristics of the subject, unlike the WS test where different cut-offs are used depending on the height and gender of the people tested (Fried et al. 2001). Classification results were accurate, with values exceeding 80 % in all cases. Similar performances were observed for both the optimal combination of PS and ES, although, as expected, the Fisher combination produced the best results. Following on from this finding, it was possible to propose a three-level decision making process from the two thresholds defined from sensitivity and specificity, as shown in Table 3. Subjects could be classified as “definitely at risk”, “definitely not at risk”, and “further examination needed”). The overall conclusion is that the BQT provides information on frailty and risk of falls, however some issues modulating this general conclusion need to be addressed.

### Population characteristics

The subjects included in the present study were patients coming to a consultation for geriatric problems, and accordingly would be expected to have a higher probability of frailty and greater fall risk. The absence of “healthy” older subjects without an appreciable fall risk could have had an effect on the performance achieved. Despite this limitation, the distribution of the values for the clinical tests and the balance scores were normally distributed, as shown for instance in Fig. 2 for the ES. The inclusion of a group of older control subjects may have modified the distribution of the variables, thereby potentially increasing the classification performance. Such a classification with additional control subjects was not possible due to the homogeneous population studied. Nevertheless, the hypothesis that the score was at a maximum for control subjects was verified with a younger subject group. None of these subjects were unable to stand on one leg for the required 15 s, or obtained an ES of <15.

### Measurements

In the present study, measurements for TUG and WS were obtained according to validated protocols by a clinician with a manual chronometer, with clinicians retaining only the integer part of the measurement in seconds. This methodology created an uncertainty between consecutive values in time, as well as an irregular distribution of the possible values on the range of WS. Such a method could be potentially critical when making a decision on the basis of a single cut-off value, for instance after a one-leg stand test. This method could be improved in two ways, either by providing an automated measurement with more precision, or by adding an uncertainty zone. The second option will be discussed in more detail below in relation to the classification results. In respect to an improved measurement system, the OS test could be performed on the BQT, with stand time calculated based on mediolateral displacement of the CoP. An automated device could also be used to measure walking speed, a prototype of which the present authors have already developed (Jaber et al. 2014).

### Empirical score versus optimized combinations

Using PS instead of the NV produced by the BQT improved the correlation with WS, even though these variables had been empirically segmented in a previous work (Duchêne and Hewson 2011). Furthermore, the results from PS were potentially better than from ES for correlation as well as for classification, which was not surprising as both models were linear and the optimization searched for the best linear weighting. However, the empirical score produced results that were not too far from the optimal ones obtained. There are two possible methods that could improve performance, especially in terms of classification:

- Refine the weight of PS by using a greater number of subjects, and then testing the performance of PS on additional subjects,
- Changing the thresholds used to attribute scores to each of the four NV used to create the PS, in order to optimize the performance of the ES. The results produced by the control population show that at the very least, the thresholds for trajectory velocity should be reassessed, something that is currently under way.

In addition to these improvements, it could be interesting to explore other ways to characterize the different phases of the weighing process, especially the stabilogram. Thus far only the most widely used variables from stabilogram analysis have been used. However, other approaches could be considered, such as taking into account the possible non-linear nature of the signal. Finally, the use of a median value for multiple tests rather than the mean would reduce the influence of any outliers.

### Clinical reference tests

The three reference tests used (WS, TUG, OS) were chosen as they are recognized for their pertinence in respect to frailty and fall risk (Fried et al. 2001; Shumway-Cook et al. 2000; van Kan et al. 2009; Vellas et al. 1997). In addition, all three provide objective results from instrumented tests. The choice of these tests is obviously an issue for discussion, as none of the tests measure the same underlying physical process as the BQT, with all three producing indirect measures of the final aim of identifying fall risk. At this point in time, there is no “Gold Standard” that could be taken as a reference for fall risk, something that would need an extensive longitudinal experiment. Such an objective is worthy of further investigation.

### Fall risk and decision making

Given the result shown in Fig. 4, there is a clear intermediate area in which there is a high probability of a bad detection or false alarm. This area occurs when ES ranges from 7 to 8 (Table 3). It follows that a three-fold decision could be made based on this zone of uncertainty:

- ES < 7: fall risk
- ES > 8: no risk of fall
- 7 ≤ ES ≤ 8: further tests are needed.

Given that ES is computed automatically, without requiring any specific learning, the BQT is well suited for monitoring community-dwelling older adults who are at the pre-frail stage, or who have recently returned home after a fall requiring hospitalization.

## Conclusion

Balance quality measured by a modified bathroom scale is correlated with standard clinical tests, which are frequently used to assess frailty or fall risk. The BQT device is very easy to use, user friendly, and fits well in the usual environment of older adults, with no difference detected when compared to a typical bathroom scale. The BQT can, therefore, be used as part of a set of tests for frailty detection, or as a stand-alone tool for balance quality assessment and ecological balance monitoring. Further investigations can be envisaged in order to refine some of the steps used to build the balance score, in particular the thresholds used to attribute scores to each of the four variables. Despite these plans, the BQT in its current form is well suited to measure balance quality and as a screening tool for fall risk.

## Appendix

Details of the four parameters calculated by the BQT are provided below:

### CoP trajectory

1. The stabilogram surface area was estimated as the product of the SD of the CoP displacement in the anteroposterior and mediolateral directions, after excluding the step on and step off segments. The surface was standardized by multiplying the surface area by 4**p**, roughly approximating an ellipse. Intuitively, this variable expresses the level of stability during the static phase, taking into account both anteroposterior and mediolateral oscillations.
2. The average velocity of the trajectory, which was computed as the sum of the lengths of the successive sample segments, divided by the stabilogram duration. This variable (equivalent to the CoP path length in a fixed time interval) is known as a relevant measure of standing balance.

### Vertical force

1. The coefficient of variation of the vertical force Fz during the stabilization stage. This parameter was calculated during a 2-s window once the subject had stepped onto the device.
2. The rise rate (defined as the average slope between 10 and 90 % of the final weight value). This variable integrates all hesitations between the first contact of the first foot with the scale and the final phase of the contact of the second foot. It specifically expresses hesitations related to moving the second foot, thus creating inflexions or even peaks in the Fz rising phase.

## References

[CR2] Berg K, Norman KE (1996). Functional assessment of balance and gait. Clin Geriatr Med.

[CR3] Berg KO (1992). Clinical and laboratory measures of postural balance in an elderly population. Arch Phys Med Rehabil.

[CR4] Berg KO (1992). Measuring balance in the elderly: validation of an instrument. Can J Public Health.

[CR5] Duchêne J, Hewson DJ (2011). Using a modified bathroom scale for long-term balance quality assessment: relevance, usability and acceptability. J Telemedi Telecare.

[CR6] Dudewicz EJ, Mishra SN (1988). Modern mathematical statistics.

[CR7] Fisher RA (1936). The use of multiple measurements in taxonomic problems. Ann Eugen.

[CR8] Fried LP (2001). Frailty in older adults: evidence for a phenotype. J Gerontol A Biol Sci Med Sci.

[CR9] Fried LP (2005). From bedside to bench: research agenda for frailty. Sci Aging Knowl Environ.

[CR10] Gill TM, Williams CS, Tinetti ME (1995). Assessing risk for the onset of functional dependence among older adults: the role of physical performance. J Am Geriatr Soc.

[CR11] Gill TM (2002). A program to prevent functional decline in physically frail, elderly persons who live at home. N Engl J Med.

[CR12] Haas BM, Burden AM (2000). Validity of weight distribution and sway measurements of the Balance Performance Monitor. Physiother Res Int.

[CR13] Hanley JA, McNeil BJ (1982). The meaning and use of the area under a Receiver Operating Characteristic (ROC) curve. Radiology.

[CR1] Jaber R et al (2014) A new device to assess gait velocity at home. In: XIII mediterranean conference on medical and biological engineering and computing 2013. Springer International Publishing

[CR14] Langley FK, Mackintosh SFH (2007). Functional balance assessment of older community dwelling adults: a systematic review of the literature. Internet J Allied Health Sci Pract.

[CR15] Mancini M, Horak FB (2010). The relevance of clinical balance assessment tools to differentiate balance deficits. Eur J Phys Rehabil Med.

[CR16] Mathias S, Nayak U, Isaacs B (1986). Balance in elderly patients: the “get-up and go” test. Arch Phys Med Rehabil.

[CR17] Mitnitski AB, Mogilner AJ, Rockwood K (2001). Accumulation of deficits as a proxy measure of aging. Sci World J.

[CR18] Montero-Odasso M (2005). Gait velocity as a single predictor of adverse events in healthy seniors aged 75 years and older. J Gerontol A Biol Sci Med Sci.

[CR19] Shumway-Cook A, Brauer S, Woollacott M (2000). Predicting the probability for falls in community-dwelling older adults using the Timed Up & Go Test. Phys Ther.

[CR20] Theou O (2011). A comparison of the relationship of 14 performance-based measures with frailty in older women. Appl Physiol Nutr Metab.

[CR21] Tinetti ME (1986). Performance-oriented assessment of mobility problems in elderly patients. J Am Geriatr Soc.

[CR22] Tinetti ME, Williams CS (1997). Falls, injuries due to falls, and the risk of admission to a nursing home. N Engl J Med.

[CR23] van Kan GA (2009). Gait speed at usual pace as a predictor of adverse outcomes in community-dwelling older people an International Academy on Nutrition and Aging (IANA) Task Force. J Nutr Health Aging.

[CR24] Vellas BJ (1997). One-leg balance is an important predictor of injurious falls in older persons. J Am Geriatr Soc.

[CR25] Vermeulen J (2012). Construct validity of a modified bathroom scale that can measure balance in elderly people. J Am Med Dir Assoc.

[CR26] Zweig MH, Campbell G (1993). Receiver-operating characteristic (ROC) plots: a fundamental evaluation tool in clinical medicine. Clin Chem.

